# 3D Characterization of corneal deformation using ultrasound speckle tracking

**DOI:** 10.1142/S1793545817420056

**Published:** 2017-09-22

**Authors:** Keyton Clayson, Elias Pavlatos, Yanhui Ma, Jun Liu

**Affiliations:** * Department of Biomedical Engineering, The Ohio State University Columbus, OH 43210, USA; † Biophysics Interdisciplinary Group, The Ohio State University Columbus, OH 43210, USA; ‡ Department of Ophthalmology and Visual Science The Ohio State University, Columbus, OH 43210, USA

**Keywords:** Ultrasound speckle tracking, cornea, inflation, 3D

## Abstract

The three-dimensional (3D) mechanical response of the cornea to intraocular pressure (IOP) elevation has not been previously reported. In this study, we use an ultrasound speckle tracking technique to measure the 3D displacements and strains within the central 5.5 mm of porcine corneas during the whole globe inflation. Inflation tests were performed on dextran-treated corneas (treated with a 10% dextran solution) and untreated corneas. The dextran-treated corneas showed an inflation response expected of a thin spherical shell, with through-thickness thinning and in-plane stretch, although the strain magnitudes exhibited a heterogeneous spatial distribution from the central to more peripheral cornea. The untreated eyes demonstrated a response consistent with swelling during experimentation, with through-thickness expansion overriding the inflation response. The average volume ratios obtained in both groups was near 1 confirming general incompressibility, but local regions of volume loss or expansion were observed. These results suggest that biomechanical measurements in 3D provide important new insight to understand the mechanical response of ocular tissues such as the cornea.

## 1. Introduction

The cornea is a highly organized transparent tissue whose shape and biomechanical properties are important for visual acuity.^1^ When this tissue is altered or disrupted during diseases such as keratoconus or refractive surgeries, the cornea's structure and function can change significantly.^2^ Although most surgical procedures improve visual acuity, undesirable changes including hyperopic shift^3^ and post-surgical ectasia^4^ can occur and may be a result of currently undetectable mechanical instability in the tissue.

Methods for measuring corneal biomechanics have emerged to identify regions of mechanical weakness or abnormality in the cornea that may not be detected by current clinical devices. Although topographic and tomographic techniques can adequately measure structural details of the cornea, characterization of corneal biomechanical properties remains challenging. Newly developed clinical devices, including the Ocular Response Analyzer (ORA) and the Corvis ST, are believed to report viscoelastic behaviors of the cornea,^5^ but linking these measurements to traditional mechanical properties has proven difficult.^6^ Current experimental techniques such as inflation testing,^7^ strip extensometry,^8,9^ and shear testing,^10^ as well as imaging techniques such as shear wave imaging,^11^ Brillouin microscopy,^12,13^ optical coherence elastography,^14^ and ultrasound speckle tracking^15^ have reported one-dimensional (1D) or two-dimensional (2D) responses, while the interactions in and out of the imaging plane (the full three-dimensional 3D response) remain largely unknown.

Our laboratory has developed and validated a high-resolution ultrasound speckle tracking method that uses non-invasive ultrasound imaging to track the displacement of ocular tissue and calculate the 3D strains caused by changes in intraocular pressure (IOP).^16,17^ Using this technique, we acquired 3D ultrasound scans near the corneal apex of whole globes during inflation. The objective of this study was to map the 3D displacements and strains during IOP increase of dextran-treated and untreated corneas to gain insights into the 3D biomechanical responses of this tissue.

## 2. Methods

Porcine globes were obtained from a local abattoir and tested within 72 h post-mortem. In the dextran-treated group (*n* = 9), whole globes were first immersed in a 10% dextran solution for 1 h to reduce corneal swelling.^18^ The anterior chamber of the globe was infused with Optisol GS (Bausch and Lomb, Rochester, NY) via a column to control IOP and minimize corneal swelling during inflation testing. In the untreated group (*n* = 8), the globes were not pre-treated with dextran and were infused with 0.9% saline.

**Fig. 1:**
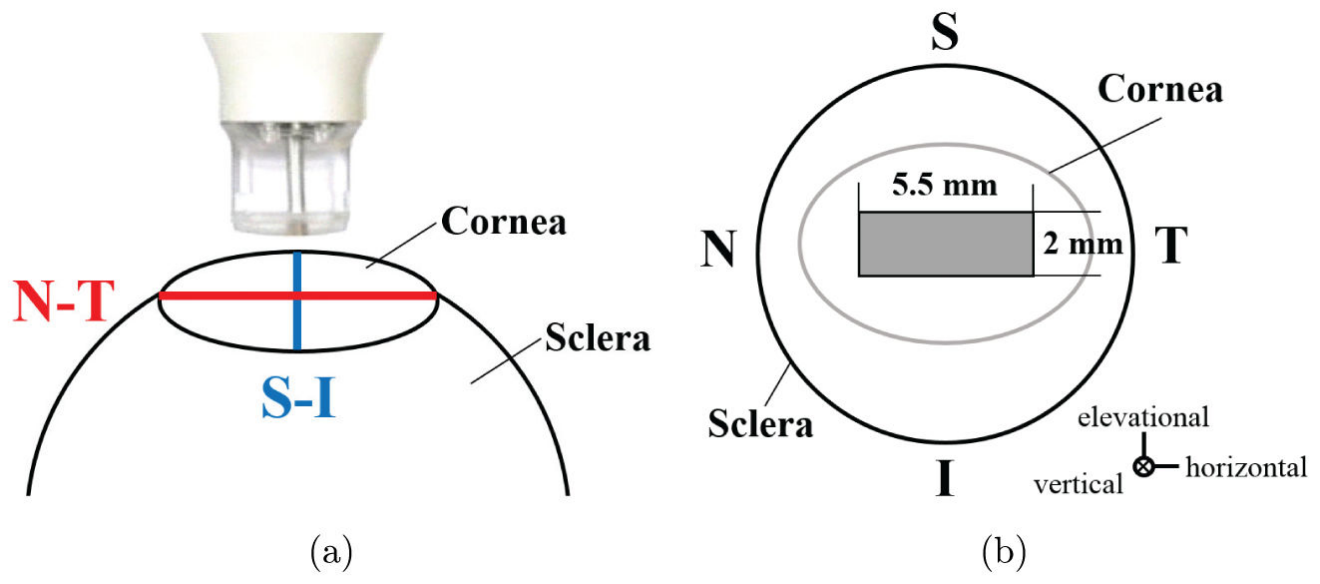
Schematics of (a) ultrasound scanning orientations and (b) 3D scanning region. (*N*: nasal, *T*: temporal, *S*: superior, *I*: inferior).

During inflation testing, all globes were secured to a custom-built holder and immersed in 0.9% saline with the cornea facing upwards. The globes were preconditioned with 25 pressure cycles from 10 mmHg to 12 mmHg and then equilibrated at a starting pressure of 10 mmHg for 1 h. Inflation tests were performed with 0.5 mmHg (untreated) or 1 mmHg (dextran-treated) steps. The small pressure steps and limited pressure range were used to allow a good possibility of successful speckle tracking and minimize unwanted tissue alterations (e.g., hydration change or decay) by completing tests within a reasonable time frame. After a 15 min equilibration time at each pressure step, a 55 MHz ultrasound probe (Vevo 660, VisualSonics Inc., Toronto) oriented along the nasal–temporal (i.e., horizontal) meridian of the cornea was used to perform a 3D ultrasound scan of the central cornea (Fig. 1(a)). Consecutive 2D B-mode images with a width of 5.5 mm were acquired in the nasal–temporal direction as the probe was translated at 14 μm steps in the superior–inferior (i.e., elevational) direction over a 2 mm distance. A stack of 288 2D images was combined to form a 3D volume centered on the corneal apex (Fig. 1(b)).

The 3D displacements, principal strains (*ε*_1_, *ε*_2_, *ε*_3_), and volume ratio at each IOP step during inflation were calculated using a cross-correlation-based speckle tracking algorithm.^16,17^ Specifically, a grid of overlapping kernels with dimensions of 434 × 76.5 × 434 *μ*m^3^ (horizontal × vertical × elevational) were defined in the scanned volume. The new location of every kernel in the tissue volume at each IOP step was determined by identifying the maximum correlation coefficient within a search window of 854 × 151.5 ×854 *μ*m^3^ centered at the original kernel location. The deformed location of the grid point was further refined using spline interpolation for sub-voxel resolution. Displacements were calculated as vectors between the kernel locations at successive IOP steps. A kernel overlap of 75% in all directions was used to achieve high spatial resolution of the strain maps.

A 3D least squares estimation method was used to obtain the displacement gradients for each kernel as follows:

(1) $$u=\frac{\partial u}{\partial x}x+\frac{\partial u}{\partial y}y+\frac{\partial u}{\partial z}z+C_{1},\;v=\frac{\partial v}{\partial x}x+\frac{\partial v}{\partial y}y+\frac{\partial v}{\partial z}z+C_{2},\;w=\frac{\partial w}{\partial x}x+\frac{\partial w}{\partial y}y+\frac{\partial w}{\partial z}z+C_{3},$$

where *u*, *v*, and w are the displacements computed from cross-correlation speckle tracking; *x*, *y*, and *z* are the kernel coordinates in the reference ultra-sound image; *C*_1_, *C*_2_, and *C*_3_ are local fitting constants; and $\frac{\partial u}{\partial x},\;\frac{\partial u}{\partial y},\;\frac{\partial u}{\partial z}\ldots$ are the displacement gradients. The displacement gradients were used to calculate the Green strain tensor for each kernel. Equations for the *e_xx_* and *e_xy_* tensor components are as follows:

(2) $$e_{xx}=\frac{\partial u}{\partial x}+\frac{1}{2}\;\left[{\left(\frac{\partial u}{\partial x}\right)}^{2}+{\left(\frac{\partial v}{\partial x}\right)}^{2}+{\left(\frac{\partial w}{\partial x}\right)}^{2}\right],\;e_{xy}=\frac{1}{2}\;\left(\frac{\partial u}{\partial y}+\frac{\partial v}{\partial x}\right)+\frac{1}{2}\;\left(\frac{\partial u}{\partial x}\frac{\partial u}{\partial y}+\frac{\partial v}{\partial x}\frac{\partial v}{\partial y}+\frac{\partial w}{\partial x}\frac{\partial w}{\partial y}\right),$$

and similar equations were used to determine the *e_yy_*, *e_zz_*, *e_yz_*, and *e_xz_* components of the tensor. The magnitudes and vector orientations of principal strains were then obtained by calculating the eigenvalues and eigenvectors of the Green strain tensor and were sorted such that *ε*_1_ *> ε*_2_ *> ε*_3_. The volume ratio for each kernel was then calculated using the principal strains as follows:

(3) $$\text{Volume}\;\text{ratio}=(1+\varepsilon_{1})(1+\varepsilon_{2})(1+\varepsilon_{3}).$$

Correlation coefficients between pressure steps were evaluated to ensure proper tracking. Two dextran-treated porcine eyes were excluded from the final data set due to unsatisfactory speckle tracking (correlation coefficients < 0.6 throughout most of the volume) caused by a weak corneal speckle pattern. 3D strain maps were visualized in Para-View (v4.4, Kitware Inc., Clifton Park, NY), whereas 2D cross-sectional displacement and strain maps and 3D strain vector orientation plots were generated in MATLAB (r2014a, The Mathworks, Inc., Natick, MA).

## 3. Results

**Fig. 2:**
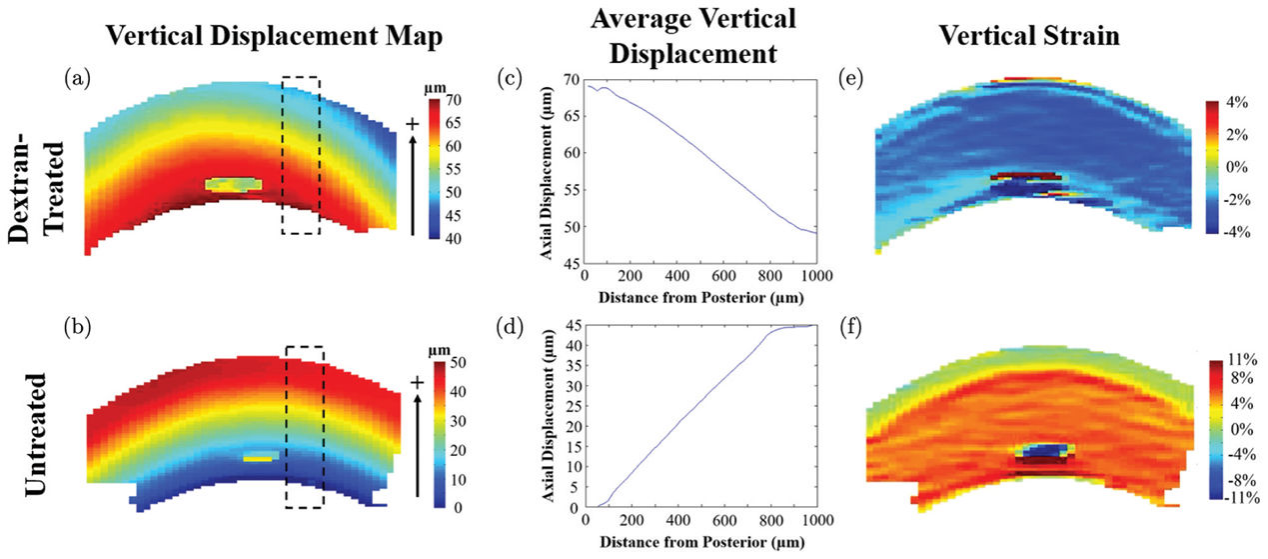
Vertical displacement (a, b) and vertical strain (e, f) plots of the central slice of representative corneas obtained in dextran-treated (a, c, e) or untreated (b, d, f) groups after inflation from 10 mmHg to 12 mmHg. The average displacement at different corneal depths (c, d) is obtained in the dashed regions of (a, b) and shows a constant displacement gradient that generates the horizontally homogenous vertical strain profiles in (e, f). Note that the irregular “spot” in the central posterior region of all images is caused by poor speckle tracking in the respective region.

Maps of vertical displacements and strains are shown for a central cross-section of representative dextran-treated and untreated corneas in Fig. 2. After inflation from 10 mmHg to 12 mmHg, dextran-treated corneas showed an upward vertical displacement with a decreasing magnitude from the posterior surface to the anterior surface (i.e., negative gradient, Figs. 2(a) and 2(c)). An opposite trend was observed in the untreated corneas, with an increasing upward displacement from the posterior to the anterior surface (i.e., positive gradient, Figs. 2(b) and 2(d)). The displacement gradient was consistent from the center to the periphery of the volume in both groups, which resulted in a consistent vertical strain response from center to periphery within the cross-section (Figs. 2(e) and 2(f)).

**Fig. 3:**
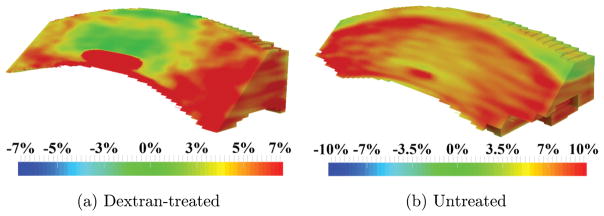
Maps of the first principal strain *ε*_1_ obtained in (a) dextran-treated, and (b) untreated globes after inflation from 10 mmHg to 12 mmHg.

Maps of the first principal strain *ε*_1_ are shown in Fig. 3. The first principal strain *ε*_1_ was positive (i.e., tensile) in both corneal groups. In the dextran-treated corneas, the first principal strain *ε*_1_ was smaller in the central region than the periphery with little to no anterior to posterior variation. In untreated corneas, there was a consistent tensional first principal strain *ε*_1_ from central to periphery (red, Fig. 3(b)), but a smaller magnitude was observed in the very anterior layer (yellow/green, Fig. 3(b)).

**Fig. 4:**
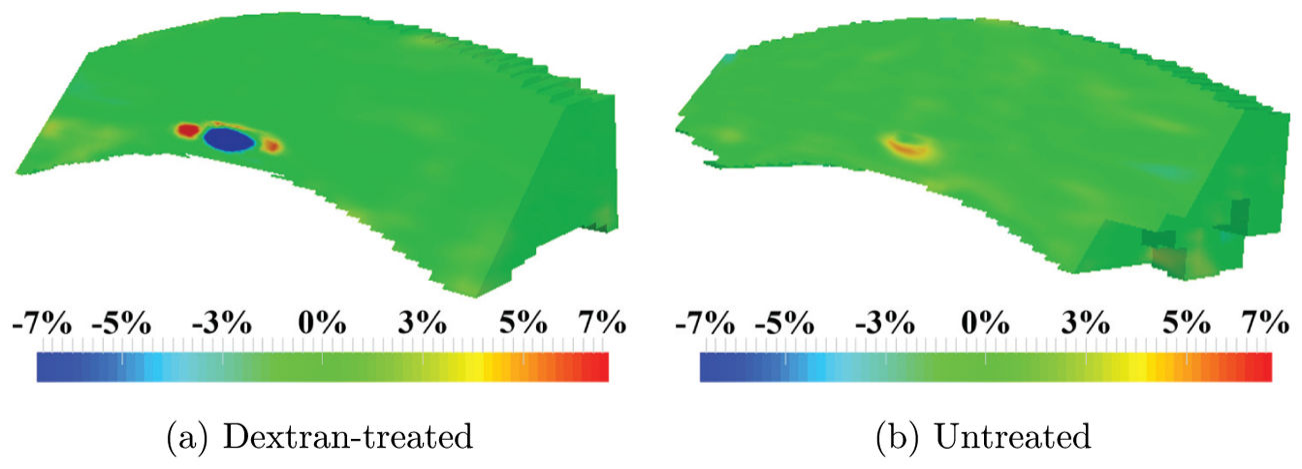
Maps of the second principal strain *ε*_2_ obtained in (a) dextran-treated, and (b) untreated globes after inflation from 10 mmHg to 12 mmHg.

Maps of the second principal strain *ε*_2_ are shown in Fig. 4. The second principal strain *ε*_2_ is defined as the intermediate value of the three principal strains, which may be compressive or tensile in nature. In both groups, the second principal *ε*_2_ had a small magnitude throughout the majority of the volume of the cornea (green, Fig. 4).

**Fig. 5:**
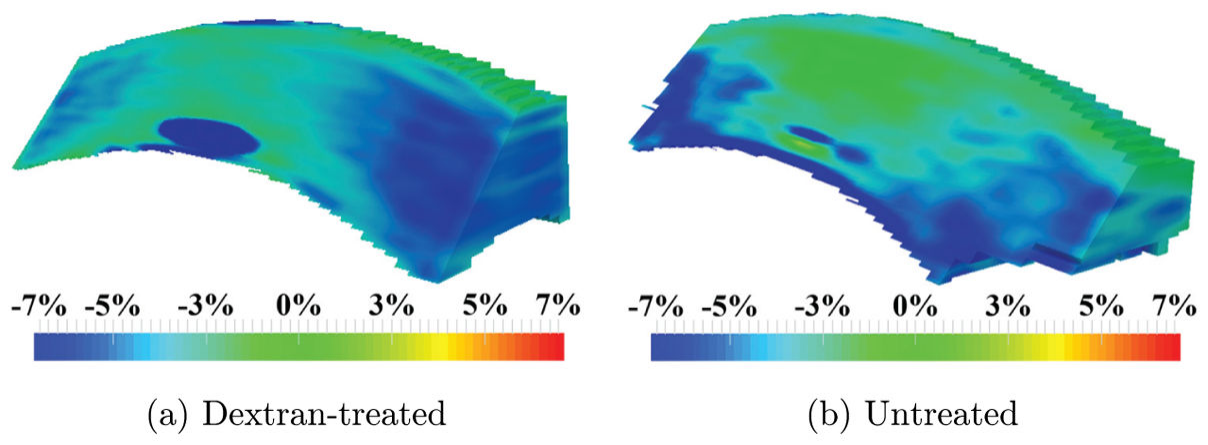
Maps of the third principal strain *ε*_3_ obtained in (a) dextran-treated, and (b) untreated globes after inflation from 10 mmHg to 12 mmHg.

Maps of the third principal strains are shown in Fig. 5. The third principal strain *ε*_3_ was compressive in nature (i.e., negative) in both groups. In dextran-treated eyes, the third principal strain *ε*_3_ was smaller in the central region than the periphery with little through-thickness variation. In untreated globes, the compressive strains were mostly found in the posterior cornea.

**Fig. 6:**
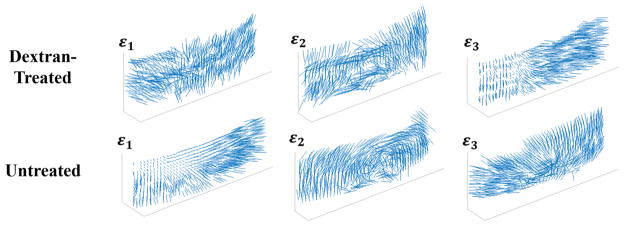
Vector plots of the orientation of the three principal strains (*ε*_1_, *ε*_2_, *ε*_3_) on a spherical surface selected from the mid-stroma of the dextran-treated and untreated corneas after inflation from 10 mmHg to 12 mmHg.

Principal strain vector plots for the dextran-treated and untreated globes are shown in Fig. 6. The first principal *ε*_1_ in dextran-treated corneas was primarily oriented in-plane, suggesting in-plane stretch during inflation. The first principal *ε*_1_ in untreated corneas was largely oriented through the corneal thickness, demonstrating expansion in thickness consistent with corneal swelling. The second principal *ε*_2_ was primarily in-plane for both groups of corneas, and in some eyes, a circumferential orientation was evident. The third principal *ε*_3_ in dextran-treated corneas showed preferential orientation through the corneal thickness, demonstrating through-thickness compression, while untreated corneas had in-plane compression primarily in the posterior region.

**Fig. 7:**
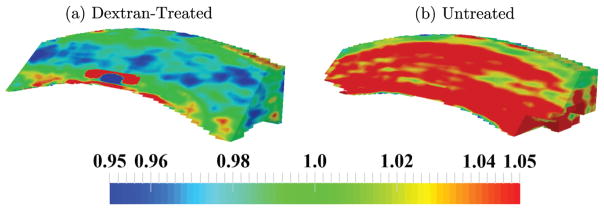
Maps of the volume ratio obtained in (a) dextran-treated, and (b) untreated globes after inflation from 10 mmHg to 12 mmHg.

3D maps of the volume ratio for both groups of corneas are presented in Fig. 7. All volume ratio values were close to 1, but regions of lower or higher values were observed. In the dextran-treated corneas, the region just below the anterior corneal surface had a volume ratio lower than 1 (blue, Fig. 7(a)), while the posterior surface contained regions with volume ratios larger than 1 (red, Fig. 7 (a)). In untreated corneas, the volume ratio was larger than 1 throughout the thickness except in the most anterior region of the cornea (Fig. 7(b)).

Average values of the three principal strains and the volume ratio for both the dextran-treated and untreated groups are presented in Table 1. For dextran-treated corneas, the third principal strain *ε*_3_ was negative and the largest in magnitude, while the first principal strain *ε*_1_ was positive and smaller in magnitude and the second principal strain *ε*_2_ was very small in magnitude. For untreated globes, the first principal strain *ε*_1_ was positive and the largest in magnitude, while the third principal strain *ε*_3_ was negative and smaller in magnitude and the second principal strain *ε*_2_ was again very small in magnitude. The volume ratio for untreated corneas was on average just above 1, while the volume ratio for dextran-treated corneas was on average slightly less than 1.

## 4. Discussion

The 3D inflation response of the cornea, i.e., the volumetric distribution of displacements and strains has not been previously reported. In this study, we used an ultrasound speckle tracking technique to map the 3D response of the central cornea in *ex vivo* porcine globes that either received dextran treatment or were untreated before testing. We found that dextran-treated corneas responded to whole globe inflation with through-thickness compression and in-plane tension, as expected for a spherical thin shell albeit with interesting regional heterogeneity. In contrast, untreated globes demonstrated primarily through-thickness expansion, which was indicative of corneal swelling during testing that overrode the inflation response. We also observed that although the average volume ratio was close to 1, the volume ratio was heterogeneous showing regional volume loss (volume ratio < 1) or expansion (volume ratio > 1) suggesting redistribution of fluid through the tissue. These results suggest that biomechanical measurements in 3D provide important new insight to understand the mechanical response of the cornea.

We observed that for dextran-treated corneas, the first principal strain *ε*_1_ was positive and primarily oriented in-plane, while the third principal *ε*_3_ was negative and primarily oriented through the thickness. This is largely consistent with the expected inflation response of a thin spherical shell with in-plane stretch and through-thickness compression. Interestingly, the central cornea showed a smaller stretch (Fig. 3(a)) and compression (Fig. 5 (a)) than the periphery, while a homogenous response would be predicted for a homogenous, isotropic thin shell. Smaller deformation in the central cornea during inflation was also reported in bovine corneas using digital image correlation.^7^ The regional difference may be an outcome of the preferred collagen fiber alignment in these corneas, as shown by wide angle X-ray scattering.^19^ Further studies are needed to verify this result.

It is of interest to note that the cross-sectional view of the vertical displacement map shown in Fig. 2 showed no difference from the central to peripheral cornea and thus generates a homogenous vertical strain profile if only the data within the cross-section was used, as in a 2D study. This outcome highlights the importance of obtaining 3D data to accurately characterize the biomechanical responses of a 3D structure.

We found that untreated corneas, although inflated at the same pressure levels as the dextran-treated group, showed a distinct mechanical profile as the positive first principal *ε*_1_ was primarily oriented through the thickness. This response is consistent with increased tissue thickness due to swelling, and appears to override any observable response caused by the pressure increase during inflation. In contrast to dextran-treated globes, the second principal *ε*_2_ vectors showed a circumferential orientation centered on the corneal apex, which appeared to be consistent with the preferred collagen fiber alignment in porcine corneas.^19^ The compressive strains observed in the posterior cornea of this group were consistent with the reported posterior stroma folding after severe swelling.^20^

Spatial variations in volume ratios were observed in both dextran-treated and untreated corneas. To our best knowledge, measurements of volume ratios in corneas have not been reported before, and almost all computational models of the cornea assume near-incompressibility (i.e., a volume ratio of 1) at each point in the tissue. In dextran-treated eyes, small regions of the posterior cornea (Fig. 7(a)) had volume ratios greater than 1, which suggests that swelling was not completely suppressed by Optisol in the posterior stroma of these eyes. The mid-stroma also contained regions with volume ratios less than 1, which may indicate fluid movement during inflation. In untreated globes, most of the tissue had a volume ratio greater than 1, suggesting active swelling and the absorption of fluid by the corneal stroma during experimentation. These results indicate that although the cornea may exhibit an overall incompressible profile, regional differences may exist that could be important for accurate modeling of the tissue responses.

There are a number of limitations of the current study. First, only a small volume of the cornea was measured, preventing quantitative analysis of regional variances such as between central and peripheral cornea. Future work will expand imaging to other regions of the cornea and anterior sclera to acquire a more complete map. In addition, future work will examine clinically relevant models, such as the depth-dependent biomechanical changes that occur after the creation of a lamellar flap or after refractive ablation in various corneal surgical procedures. Second, tissue deformation within a limited pressure range near the lower end of physiological IOP (10 mmHg to 12 mmHg) was measured in this study. As the choice of the small range was based on practical considerations in this pilot study (e.g., avoiding excessively long experimental time), our future work will aim to establish better corneal hydration control and measure 3D corneal deformation over larger pressure ranges. Third, Optisol immersion of dextran-treated globes did not fully control corneal swelling, especially posterior swelling during experimentation and therefore the inflation response observed in the treated corneas was confounded by swelling to some extent. In comparison to the untreated corneas, however, Optisol immersion mitigated the effects of uncontrolled swelling in the dextran-treated globes. Future studies are needed to better address the swelling issue in *ex vivo* corneal experimentation by either developing more effective hydration control media or quantifying the separate effects of swelling pressure and inflation response.

In summary, this study demonstrated the feasibility of using ultrasound speckle tracking for 3D mapping of corneal deformation. Our results showed distinct biomechanical responses in dextran-treated and untreated corneas during IOP increase, which indicates that the hydration state strongly influences the mechanical outcome observed in *ex vivo* experiments. 3D characterization of corneal biomechanical responses may help to better identify the biomechanical factors involved in corneal pathophysiology and surgical outcomes.

## Figures and Tables

**Table 1:** Average principal strains (*ε*_1_, *ε*_2_, *ε*_3_,) and volume ratio in porcine cornea.

	Pressure (mmHg)	*ε*_1_(%)	*ε*_2_(%)	*ε*_3_(%)	Volume ratio
Dextran-treated (*n* = 7)	11	1.30 ± 0.61	0.13 ± 0.52	−2.06 ± 0.71	0.993 ± 0.014
	12	2.35 ± 1.05	0.24 ± 0.85	−3.65 ± 1.27	0.987 ± 0.021
Untreated (*n* = 8)	11	3.79 ± 1.20	0.28 ± 0.60	−1.30 ± 0.78	1.026 ± 0.016
	12	7.86 ± 2.05	0.46 ± 0.95	−2.42 ± 1.39	1.054 ± 0.027
